# Crystal structure of [μ_2_-1,1′-bis­(di­phenyl­phos­phanyl)ferrocene-κ^2^ *P*:*P*′]bis­[(pyrrolidine-1-carbo­dithioato-κ*S*)gold(I)]

**DOI:** 10.1107/S2056989015016382

**Published:** 2015-09-12

**Authors:** Yee Seng Tan, Edward R. T. Tiekink

**Affiliations:** a Department of Chemistry, University of Malaya, 50603 Kuala Lumpur, Malaysia; b Centre for Chemical Crystallography, Faculty of Science and Technology, Sunway University, 47500 Bandar Sunway, Selangor Darul Ehsan, Malaysia

**Keywords:** crystal structure, gold(I), phosphane, di­thio­carbamate

## Abstract

The asymmetric unit of the title compound, {(C_34_H_28_FeP_2_)[Au(C_5_H_8_NS_2_)]_2_}, comprises half a mol­ecule, with the full mol­ecule being generated by the application of a centre of inversion. The independent Au^I^ atom is coordinated by thiol­ate S and phosphane P atoms that define an approximate linear geometry [S—Au—P = 169.35 (3)°]. The deviation from the ideal linear is traced to the close approach of the (intra­molecular) non-coordinating thione S atom [Au⋯S = 3.1538 (8) Å]. Supra­molecular layers parallel to (100) feature in the crystal packing, being sustained by phen­yl–thione C—H⋯S inter­actions, with the non-coordinating thione S atom in the role of a dual acceptor. Layers stack with no specific inter­actions between them.

## Abstract 2

The centrosymmetric mol­ecule features a linearly coordinated Au^I^ atom within an S(di­thio­carbamate) and P(phosphane) donor set.

## Chemical context   

Investigations into the potential anti-cancer activity of phosphanegold(I) di­thio­carbamates, *R*_3_PAu(S_2_CN*R*′_2_), date back over a decade (de Vos *et al.*, 2004 ▸; Vergara *et al.*, 2007 ▸; Jamaludin *et al.*, 2013 ▸). These investigations are complemented by the recently reported impressive anti-microbial activity for this class of compound (Sim *et al.*, 2014 ▸) whereby *R*_3_PAu[S_2_CN(^*i*^Pr)CH_2_CH_2_OH], *R* = Ph and Cy, exhibited specific activity against Gram-positive bacteria while the *R* = Et derivative displayed broad-range activity against both Gram-positive and Gram-negative bacteria. Motivated by observations that 1,1′-bis­(di­phenyl­phosphan­yl)ferrocene (dppf) derivatives also possess biological activity (Ornelas, 2011 ▸; Braga & Silva, 2013 ▸), it was thought of inter­est to couple dppf with Au^I^ di­thio­carbamates. This led to the isolation of the broadly insoluble title compound, dppf{Au[S_2_CN(CH_2_)_4_]}_2_, (I), which was subjected to a crystal structure determination. The results of this study are reported herein along with a comparison to related species.

## Structural commentary   

**Figure 1:**
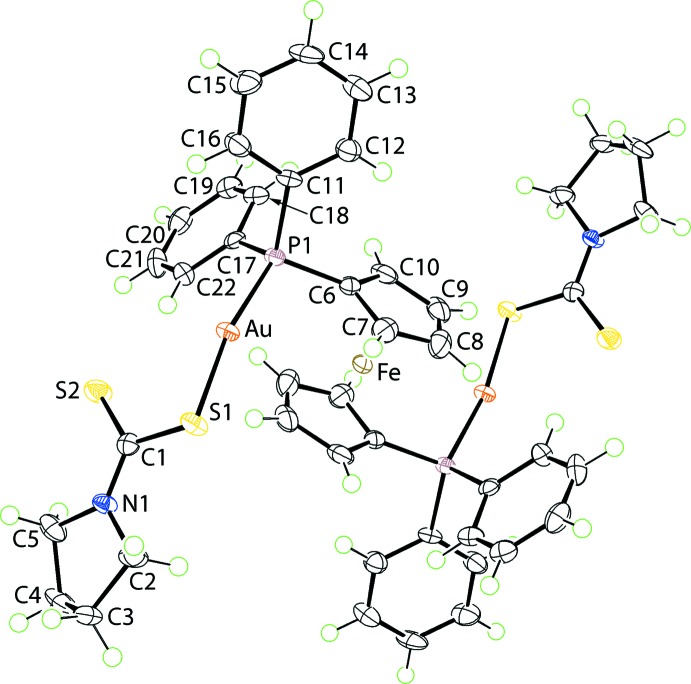
The mol­ecular structure of (I), showing the atom-labelling scheme and displacement ellipsoids at the 70% probability level. Unlabelled atoms are related by the symmetry operation (−*x* + 1, −*y*, −*z* + 2).

The Fe^II^ atom in dppf{Au[S_2_CN(CH_2_)_4_]}_2_, (I), is located on a centre of inversion, Fig. 1 ▸. The Au^I^ central atom exists in the anti­cipated linear geometry defined by thiol­ate-S and phosphane-P atoms. The Au—S1 bond length is considerably longer than the Au—P1 bond, *i.e*. 2.3378 (8) *cf*. 2.2580 (8) Å. The di­thio­carbamate ligand is orientated to place the S2 atom in close proximity to the Au^I^ atom. However, the resulting intra­molecular Au⋯S2 inter­action is long at 3.1538 (8) Å, consistent with a monodentate mode of coordination for the di­thio­carbamate ligand. The pattern of C1—S1, S2 bond lengths supports this conclusion in that the strongly bound S1 atom forms a longer, *i.e*. weaker, C1—S1 bond [1.757 (3) Å] *cf*. with C1—S2 of 1.689 (3) Å. Nevertheless, the close approach of the S2 atom to the Au^I^ central atom is correlated with the deviation from the ideal linear geometry, *i.e*. S1—Au—P1 is 169.35 (3)°. 
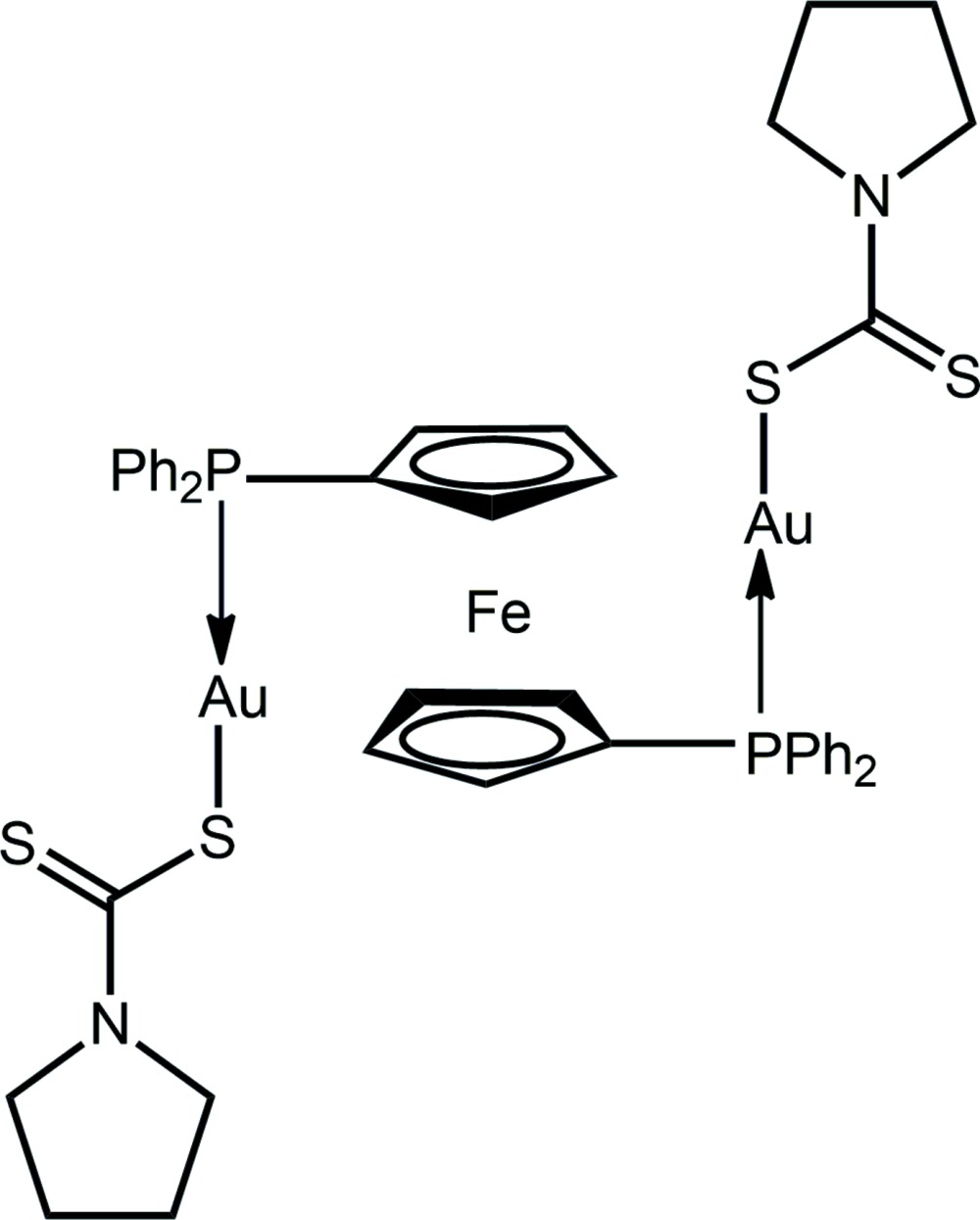


Similar features are noted in related structures as outlined below in the *Database survey*. The pyrrolidine ring is twisted about the C2—C3 bond. Owing to being located on a centre of inversion, the Fe^II^ atom is equidistant from the ring centroids of the Cp rings [Fe⋯*Cg*, *Cg* ^i^ = 1.6566 (13) Å] and the *Cg*—Fe—*Cg* ^i^ angle is constrained by symmetry to be 180°; symmetry operation (i): 1 − *x*, −*y*, 2 − *z*. Again, from symmetry, the Cp rings have a staggered relationship.

## Supra­molecular features   

**Figure 2:**
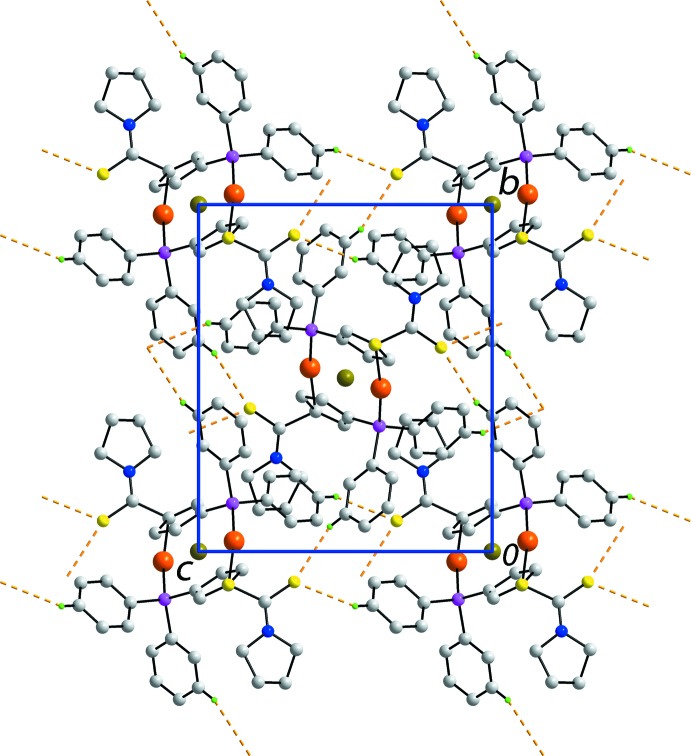
A view of the supra­molecular layer in the *bc* plane sustained by phen­yl–thione C—H⋯S inter­actions, shown as orange dashed lines. H atoms not involved in inter­molecular inter­actions have been omitted for clarity.

**Figure 3:**
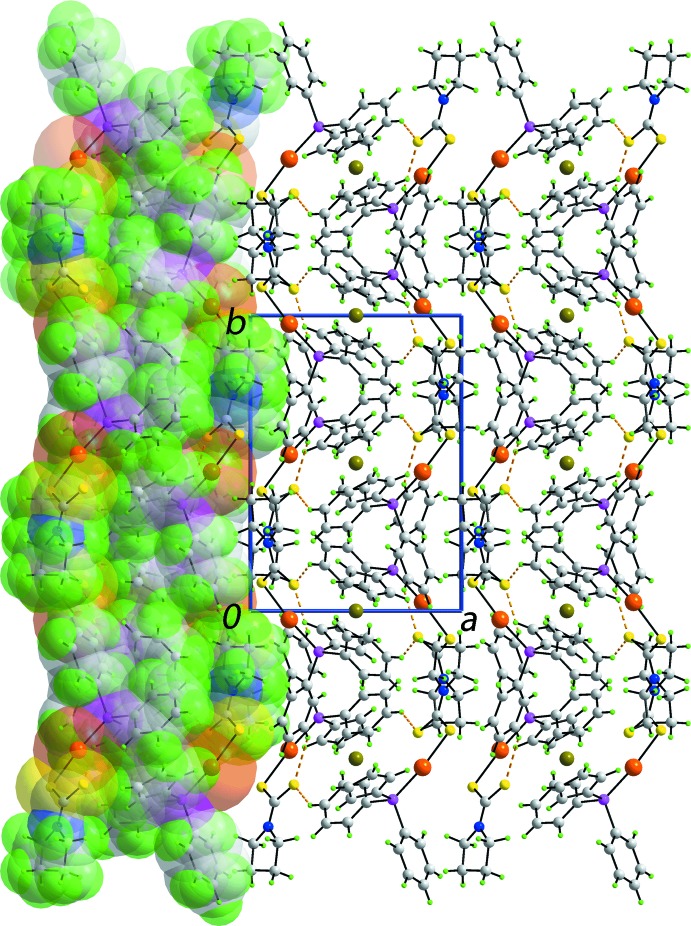
Unit-cell contents shown in projection down the *c* axis, showing the stacking of supra­molecular layers. The phen­yl–thione C—H⋯S inter­actions are shown as orange dashed lines. One layer is shown in space-filling mode.

In the crystal packing, the most prominent inter­actions are of the type C—H⋯S. Data for the phenyl-C—H⋯S(thione) inter­actions are collected in Table 1 ▸. These inter­actions, involving the dual acceptor S2 atom, serve to assemble mol­ecules into supra­molecular layers in the *bc* plane, Fig. 2 ▸. The thickness of each layer corresponds to the length of the *a* axis, *i.e*. 10.9635 (4) Å, and the layers stack along this axis with no directional inter­actions between them, Fig. 3 ▸.

## Database survey   

It has been approximately 40 years since the first report of a structure related to (I), *i.e*. Ph_3_PAu(S_2_CNEt_2_), by Wijnhoven *et al.* (1972 ▸). This serves as the archetype for approximately 20 other neutral phosphanegold(I) di­thio­carbamate structures in the crystallographic literature (Groom & Allen, 2014 ▸), each having a more or less linear P—Au—S arrangement. There are two structures containing the pyrrolinedi­thio­carbamate ligand, as in (I), but with phosphane ligands Ph_3_P [(II); Ho & Tiekink, 2004 ▸] and Cy_3_P [(III); Ho & Tiekink, 2002 ▸]. From the data collated for (I)–(III) in Table 2 ▸, it is evident that the basic structural features in all three compounds are similar. There is also a closely related dppf-type structure whereby a methyl­ene bridge has been inserted between one P atom and the Cp ring, *i.e*. (Ph_2_PCH_2_C_5_H_4_FeC_5_H_4_PPh_2_)[Au(S_2_CNEt_2_)]_2_·2CHCl_3_, [(IV); Štěpnička & Císařová, 2012 ▸]. In this analogue of (I), the Fe^II^ atom is in a general position. While the Au_2_P_2_ entity in (IV) remains approximately co-planar, as is crystallo­graphically imposed in (I), *i.e*. the Au—P⋯P—Au pseudo torsion angle is 161.82 (5)°, the Au^I^ atoms lie approximately to the same side of the mol­ecule as opposed to the strictly *anti* conformation found in (I). As seen in Table 2 ▸, the selected geometric parameters in (I) and (IV) are comparable. Despite having the shortest intra­molecular Au⋯S2 contact in (IV), the deviation of the S—Au—P angle from linearity is not the greatest in this structure.

## Synthesis and crystallization   

Two solutions were prepared. Firstly, a solution of the sodium salt of pyrrolidine di­thio­carbamate (Aldrich, 1.6 mmol) was prepared by dissolving this (0.2628 g) in methanol (25 ml). A second solution containing [1,1′-bis­(di­phenyl­phosphan­yl)ferrocene]bis­[chlorido­gold(I)] (synthesized by the reduction of KAuCl_4_ by Na_2_SO_3_ followed by the addition of a stoichiometric amount of 1,1′-bis­(di­phenyl­phosphan­yl)ferrocene; 0.8154 g, 0.8 mmol) was prepared by dissolution in di­chloro­methane (75 ml). The solution containing the di­thio­carbamate salt was added to the gold precursor solution. The resulting mixture was stirred for 3 h at room condition and then filtered. After a week of slow evaporation in a refrigerator, some dark-yellow blocks appeared that were characterized crystallographically. M. p. 378–379 K. IR (cm^−1^): 1435 *s* ν(C—N); 1152 *m*, 996 *m* ν(C—S).

## Refinement   

Crystal data, data collection and structure refinement details are summarized in Table 3 ▸. Carbon-bound H-atoms were placed in calculated positions (C—H = 0.95–0.99 Å) and were included in the refinement in the riding-model approximation, with *U*_iso_(H) set to 1.2*U*_eq_(C). The maximum and minimum residual electron density peaks of 1.57 and 1.11 e Å^−3^, respectively, were located 0.92 and 0.79 Å from the Au atom.

## Supplementary Material

Crystal structure: contains datablock(s) I, global. DOI: 10.1107/S2056989015016382/vn2097sup1.cif

Structure factors: contains datablock(s) I. DOI: 10.1107/S2056989015016382/vn2097Isup2.hkl

CCDC reference: 1421954

Additional supporting information:  crystallographic information; 3D view; checkCIF report

## Figures and Tables

**Table 1:** Hydrogen-bond geometry (, )

*D*H*A*	*D*H	H*A*	*D* *A*	*D*H*A*
C13H13S2^i^	0.95	2.86	3.680(3)	144
C20H20S2^ii^	0.95	2.84	3.628(3)	141

Symmetry codes: (i) 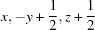; (ii) 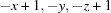.

**Table 2:** Geometric details (, ) for (I) and related literature structures

Structure	AuS	AuP	SAuP	AuS2	CSD Refcode*^*a*^*	Reference
(I)	2.3378(8)	2.2580(8)	169.35(3)	3.1538(8)		This work
(II)	2.3333(11)	2.2447(10)	173.82(4)	3.0440(10)	AYIYAI	Ho Tiekink (2004 ▸)
(III)	2.3256(16)	2.2547(15)	176.55(5)	3.1067(17)	XUMRIG	Ho Tiekink (2002 ▸)
(IV)	2.3365(11)	2.2495(10)	171.98(3)	3.0472(10)	GICZAV	tpnika Csaov (2012 ▸)
	2.3559(8)	2.2459(8)	172.12(3)	2.9178(12)		

Reference: (*a*) Groom Allen (2014 ▸).

**Table 3:** Experimental details

Crystal data	
Chemical formula	[Au_2_Fe(C_5_H_8_NS_2_)_2_(C_34_H_28_P_2_)]
*M*_r_	1240.77
Crystal system, space group	Monoclinic, *P*2_1_/*c*
Temperature (K)	100
*a*, *b*, *c* ()	10.9635(4), 14.9720(5), 13.0087(4)
()	102.977(3)
*V* (^3^)	2080.78(12)
*Z*	2
Radiation type	Mo *K*
(mm^1^)	7.69
Crystal size (mm)	0.20 0.20 0.20

Data collection	
Diffractometer	Agilent SuperNova Dual diffractometer with an Atlas detector
Absorption correction	Multi-scan (*CrysAlis PRO*; Agilent, 2014 ▸)
*T*_min_, *T*_max_	0.294, 1.000
No. of measured, independent and observed [*I* > 2(*I*)] reflections	24384, 4777, 4363
*R*_int_	0.048
(sin /)_max_ (^1^)	0.650

Refinement	
*R*[*F* ^2^ > 2(*F* ^2^)], *wR*(*F* ^2^), *S*	0.022, 0.051, 1.06
No. of reflections	4777
No. of parameters	250
H-atom treatment	H-atom parameters constrained
_max_, _min_ (e ^3^)	1.57, 1.11

Computer programs: *CrysAlis PRO* (Agilent, 2014 ▸), *SHELXS97* (Sheldrick, 2008 ▸), *SHELXL2014* (Sheldrick, 2015 ▸), *ORTEP-3 for Windows* (Farrugia, 2012 ▸), *DIAMOND* (Brandenburg, 2006 ▸) and *publCIF* (Westrip, 2010 ▸).

## supplementary crystallographic information

### Crystal data

**Table:**

[Au_2_Fe(C_5_H_8_NS_2_)_2_(C_34_H_28_P_2_)]	*F*(000) = 1200
*M**_r_* = 1240.77	*D*_x_ = 1.980 Mg m^−^^3^
Monoclinic, *P*2_1_/*c*	Mo *K*α radiation, λ = 0.71073 Å
*a* = 10.9635 (4) Å	Cell parameters from 10685 reflections
*b* = 14.9720 (5) Å	θ = 3.5–30.2°
*c* = 13.0087 (4) Å	µ = 7.69 mm^−^^1^
β = 102.977 (3)°	*T* = 100 K
*V* = 2080.78 (12) Å^3^	Block, dark-yellow
*Z* = 2	0.20 × 0.20 × 0.20 mm

### Data collection

**Table:**

Agilent SuperNova Dual diffractometer with an Atlas detector	4777 independent reflections
Radiation source: SuperNova (Mo) X-ray Source	4363 reflections with *I* > 2σ(*I*)
Mirror monochromator	*R*_int_ = 0.048
Detector resolution: 10.4041 pixels mm^-1^	θ_max_ = 27.5°, θ_min_ = 3.1°
ω scan	*h* = −14→14
Absorption correction: multi-scan (*CrysAlis PRO*; Agilent, 2014)	*k* = −19→19
*T*_min_ = 0.294, *T*_max_ = 1.000	*l* = −16→16
24384 measured reflections	

### Refinement

**Table:**

Refinement on *F*^2^	0 restraints
Least-squares matrix: full	Hydrogen site location: inferred from neighbouring sites
*R*[*F*^2^ > 2σ(*F*^2^)] = 0.022	H-atom parameters constrained
*wR*(*F*^2^) = 0.051	*w* = 1/[σ^2^(*F*_o_^2^) + (0.0208*P*)^2^ + 0.4001*P*] where *P* = (*F*_o_^2^ + 2*F*_c_^2^)/3
*S* = 1.06	(Δ/σ)_max_ = 0.002
4777 reflections	Δρ_max_ = 1.57 e Å^−^^3^
250 parameters	Δρ_min_ = −1.11 e Å^−^^3^

### Special details

**Table:**

Geometry. All esds (except the esd in the dihedral angle between two l.s. planes) are estimated using the full covariance matrix. The cell esds are taken into account individually in the estimation of esds in distances, angles and torsion angles; correlations between esds in cell parameters are only used when they are defined by crystal symmetry. An approximate (isotropic) treatment of cell esds is used for estimating esds involving l.s. planes.

### Fractional atomic coordinates and isotropic or equivalent isotropic displacement parameters (Å^2^)

**Table:**

	*x*	*y*	*z*	*U*_iso_*/*U*_eq_	
Au	0.81601 (2)	0.02934 (2)	0.87927 (2)	0.01301 (5)	
Fe	0.5000	0.0000	1.0000	0.01445 (13)	
S1	0.94626 (7)	−0.09602 (5)	0.89801 (6)	0.01689 (16)	
S2	0.79416 (8)	−0.08799 (5)	0.67459 (6)	0.02172 (18)	
P1	0.67514 (7)	0.13829 (5)	0.88458 (6)	0.01219 (15)	
N1	0.9156 (2)	−0.23029 (16)	0.76400 (18)	0.0145 (5)	
C1	0.8858 (3)	−0.1454 (2)	0.7746 (2)	0.0139 (6)	
C2	0.9848 (3)	−0.2871 (2)	0.8506 (2)	0.0203 (7)	
H2A	0.9382	−0.2934	0.9072	0.024*	
H2B	1.0686	−0.2619	0.8812	0.024*	
C3	0.9954 (3)	−0.3769 (2)	0.7971 (3)	0.0232 (7)	
H3A	1.0746	−0.3806	0.7727	0.028*	
H3B	0.9924	−0.4270	0.8461	0.028*	
C4	0.8842 (3)	−0.3795 (2)	0.7050 (3)	0.0245 (7)	
H4A	0.8084	−0.4004	0.7273	0.029*	
H4B	0.9001	−0.4194	0.6488	0.029*	
C5	0.8690 (3)	−0.2827 (2)	0.6669 (2)	0.0206 (7)	
H5A	0.9193	−0.2708	0.6140	0.025*	
H5B	0.7802	−0.2688	0.6356	0.025*	
C6	0.5997 (3)	0.1135 (2)	0.9904 (2)	0.0141 (6)	
C7	0.6606 (3)	0.0625 (2)	1.0801 (2)	0.0211 (7)	
H7	0.7432	0.0391	1.0924	0.025*	
C8	0.5766 (4)	0.0528 (2)	1.1475 (2)	0.0268 (8)	
H8	0.5932	0.0220	1.2130	0.032*	
C9	0.4649 (3)	0.0963 (2)	1.1010 (3)	0.0254 (8)	
H9	0.3926	0.0996	1.1296	0.030*	
C10	0.4771 (3)	0.1346 (2)	1.0040 (2)	0.0202 (7)	
H10	0.4152	0.1682	0.9568	0.024*	
C11	0.7323 (3)	0.25218 (19)	0.9075 (2)	0.0131 (6)	
C12	0.7027 (3)	0.3055 (2)	0.9856 (2)	0.0187 (7)	
H12	0.6518	0.2826	1.0298	0.022*	
C13	0.7478 (3)	0.3923 (2)	0.9990 (3)	0.0231 (7)	
H13	0.7283	0.4286	1.0531	0.028*	
C14	0.8207 (3)	0.4265 (2)	0.9344 (3)	0.0241 (7)	
H14	0.8513	0.4860	0.9442	0.029*	
C15	0.8494 (3)	0.3739 (2)	0.8554 (3)	0.0245 (7)	
H15	0.8993	0.3972	0.8107	0.029*	
C16	0.8048 (3)	0.2874 (2)	0.8419 (3)	0.0205 (7)	
H16	0.8238	0.2515	0.7874	0.025*	
C17	0.5506 (3)	0.1478 (2)	0.7674 (2)	0.0133 (6)	
C18	0.4616 (3)	0.2152 (2)	0.7586 (2)	0.0170 (6)	
H18	0.4699	0.2595	0.8120	0.020*	
C19	0.3606 (3)	0.2183 (2)	0.6723 (2)	0.0191 (7)	
H19	0.2980	0.2629	0.6680	0.023*	
C20	0.3520 (3)	0.1557 (2)	0.5924 (2)	0.0214 (7)	
H20	0.2837	0.1579	0.5329	0.026*	
C21	0.4414 (3)	0.0906 (2)	0.5986 (2)	0.0222 (7)	
H21	0.4355	0.0488	0.5428	0.027*	
C22	0.5405 (3)	0.0857 (2)	0.6862 (2)	0.0175 (6)	
H22	0.6015	0.0399	0.6907	0.021*	

### Atomic displacement parameters (Å^2^)

**Table:**

	*U*^11^	*U*^22^	*U*^33^	*U*^12^	*U*^13^	*U*^23^
Au	0.01259 (7)	0.00937 (7)	0.01715 (7)	0.00067 (4)	0.00346 (5)	−0.00134 (4)
Fe	0.0201 (3)	0.0098 (3)	0.0150 (3)	−0.0028 (3)	0.0070 (2)	−0.0025 (2)
S1	0.0171 (4)	0.0131 (4)	0.0180 (4)	0.0029 (3)	−0.0011 (3)	−0.0037 (3)
S2	0.0292 (5)	0.0162 (4)	0.0168 (4)	0.0070 (3)	−0.0012 (3)	0.0001 (3)
P1	0.0129 (4)	0.0093 (4)	0.0148 (3)	0.0002 (3)	0.0038 (3)	−0.0006 (3)
N1	0.0164 (13)	0.0101 (13)	0.0159 (12)	−0.0008 (10)	0.0011 (10)	−0.0027 (10)
C1	0.0117 (15)	0.0142 (16)	0.0160 (14)	−0.0017 (12)	0.0040 (11)	0.0003 (12)
C2	0.0243 (18)	0.0133 (16)	0.0220 (16)	0.0060 (14)	0.0025 (13)	0.0023 (13)
C3	0.032 (2)	0.0107 (16)	0.0297 (17)	0.0047 (14)	0.0126 (14)	0.0027 (13)
C4	0.0259 (19)	0.0113 (16)	0.0380 (19)	−0.0026 (14)	0.0111 (15)	−0.0110 (14)
C5	0.0192 (17)	0.0177 (17)	0.0247 (16)	−0.0033 (14)	0.0043 (13)	−0.0101 (13)
C6	0.0175 (16)	0.0097 (15)	0.0159 (14)	−0.0034 (12)	0.0056 (12)	−0.0016 (11)
C7	0.0257 (19)	0.0187 (17)	0.0166 (15)	−0.0066 (14)	−0.0001 (13)	−0.0019 (13)
C8	0.041 (2)	0.0240 (18)	0.0164 (16)	−0.0154 (17)	0.0084 (15)	−0.0058 (14)
C9	0.035 (2)	0.0177 (18)	0.0297 (17)	−0.0134 (15)	0.0205 (15)	−0.0107 (14)
C10	0.0226 (17)	0.0105 (16)	0.0306 (17)	−0.0002 (13)	0.0124 (14)	−0.0038 (13)
C11	0.0111 (15)	0.0078 (14)	0.0189 (14)	0.0008 (12)	0.0002 (11)	−0.0008 (11)
C12	0.0199 (17)	0.0158 (17)	0.0211 (15)	−0.0014 (13)	0.0061 (13)	−0.0016 (12)
C13	0.0233 (18)	0.0165 (17)	0.0302 (17)	0.0005 (14)	0.0076 (14)	−0.0072 (14)
C14	0.0231 (18)	0.0093 (16)	0.041 (2)	−0.0039 (14)	0.0095 (15)	−0.0031 (14)
C15	0.0226 (18)	0.0179 (18)	0.0370 (19)	−0.0027 (14)	0.0153 (15)	0.0029 (14)
C16	0.0222 (18)	0.0153 (17)	0.0259 (16)	0.0003 (14)	0.0095 (13)	−0.0039 (13)
C17	0.0150 (15)	0.0108 (15)	0.0151 (13)	−0.0017 (12)	0.0054 (11)	0.0033 (11)
C18	0.0177 (16)	0.0116 (16)	0.0217 (15)	−0.0010 (12)	0.0045 (12)	0.0021 (12)
C19	0.0177 (17)	0.0170 (17)	0.0230 (16)	0.0002 (13)	0.0055 (13)	0.0038 (13)
C20	0.0174 (17)	0.0299 (19)	0.0155 (14)	−0.0050 (15)	0.0007 (12)	0.0041 (13)
C21	0.0252 (18)	0.0285 (19)	0.0137 (14)	−0.0022 (15)	0.0058 (13)	−0.0049 (13)
C22	0.0206 (17)	0.0159 (16)	0.0175 (14)	0.0019 (13)	0.0074 (12)	−0.0018 (12)

### Geometric parameters (Å, º)

**Table:**

Au—P1	2.2580 (8)	C6—C7	1.430 (4)
Au—S1	2.3378 (8)	C6—C10	1.430 (4)
Fe—C10^i^	2.033 (3)	C7—C8	1.414 (5)
Fe—C10	2.033 (3)	C7—H7	0.9500
Fe—C6^i^	2.039 (3)	C8—C9	1.399 (5)
Fe—C6	2.039 (3)	C8—H8	0.9500
Fe—C9	2.044 (3)	C9—C10	1.420 (4)
Fe—C9^i^	2.044 (3)	C9—H9	0.9500
Fe—C7^i^	2.059 (3)	C10—H10	0.9500
Fe—C7	2.059 (3)	C11—C12	1.386 (4)
Fe—C8	2.071 (3)	C11—C16	1.395 (4)
Fe—C8^i^	2.071 (3)	C12—C13	1.388 (4)
S1—C1	1.757 (3)	C12—H12	0.9500
S2—C1	1.689 (3)	C13—C14	1.382 (5)
P1—C6	1.796 (3)	C13—H13	0.9500
P1—C17	1.809 (3)	C14—C15	1.386 (5)
P1—C11	1.818 (3)	C14—H14	0.9500
N1—C1	1.327 (4)	C15—C16	1.381 (5)
N1—C2	1.478 (4)	C15—H15	0.9500
N1—C5	1.478 (4)	C16—H16	0.9500
C2—C3	1.531 (4)	C17—C18	1.390 (4)
C2—H2A	0.9900	C17—C22	1.394 (4)
C2—H2B	0.9900	C18—C19	1.389 (4)
C3—C4	1.506 (5)	C18—H18	0.9500
C3—H3A	0.9900	C19—C20	1.387 (4)
C3—H3B	0.9900	C19—H19	0.9500
C4—C5	1.528 (5)	C20—C21	1.371 (5)
C4—H4A	0.9900	C20—H20	0.9500
C4—H4B	0.9900	C21—C22	1.388 (4)
C5—H5A	0.9900	C21—H21	0.9500
C5—H5B	0.9900	C22—H22	0.9500
			
P1—Au—S1	169.35 (3)	C5—C4—H4B	110.9
C10^i^—Fe—C10	180.00 (19)	H4A—C4—H4B	109.0
C10^i^—Fe—C6^i^	41.11 (12)	N1—C5—C4	103.6 (2)
C10—Fe—C6^i^	138.89 (12)	N1—C5—H5A	111.0
C10^i^—Fe—C6	138.89 (12)	C4—C5—H5A	111.0
C10—Fe—C6	41.11 (12)	N1—C5—H5B	111.0
C6^i^—Fe—C6	180.0	C4—C5—H5B	111.0
C10^i^—Fe—C9	139.24 (12)	H5A—C5—H5B	109.0
C10—Fe—C9	40.76 (12)	C7—C6—C10	107.3 (3)
C6^i^—Fe—C9	111.54 (12)	C7—C6—P1	121.7 (2)
C6—Fe—C9	68.46 (12)	C10—C6—P1	131.0 (2)
C10^i^—Fe—C9^i^	40.76 (12)	C7—C6—Fe	70.32 (18)
C10—Fe—C9^i^	139.24 (13)	C10—C6—Fe	69.22 (17)
C6^i^—Fe—C9^i^	68.46 (12)	P1—C6—Fe	124.38 (15)
C6—Fe—C9^i^	111.54 (12)	C8—C7—C6	108.1 (3)
C9—Fe—C9^i^	180.0	C8—C7—Fe	70.42 (19)
C10^i^—Fe—C7^i^	68.50 (14)	C6—C7—Fe	68.85 (17)
C10—Fe—C7^i^	111.50 (13)	C8—C7—H7	125.9
C6^i^—Fe—C7^i^	40.83 (12)	C6—C7—H7	125.9
C6—Fe—C7^i^	139.17 (12)	Fe—C7—H7	126.4
C9—Fe—C7^i^	112.49 (14)	C9—C8—C7	108.2 (3)
C9^i^—Fe—C7^i^	67.51 (14)	C9—C8—Fe	69.09 (18)
C10^i^—Fe—C7	111.50 (13)	C7—C8—Fe	69.53 (18)
C10—Fe—C7	68.50 (14)	C9—C8—H8	125.9
C6^i^—Fe—C7	139.17 (12)	C7—C8—H8	125.9
C6—Fe—C7	40.83 (12)	Fe—C8—H8	127.1
C9—Fe—C7	67.51 (14)	C8—C9—C10	109.0 (3)
C9^i^—Fe—C7	112.49 (14)	C8—C9—Fe	71.15 (19)
C7^i^—Fe—C7	180.0	C10—C9—Fe	69.22 (17)
C10^i^—Fe—C8	112.02 (14)	C8—C9—H9	125.5
C10—Fe—C8	67.98 (14)	C10—C9—H9	125.5
C6^i^—Fe—C8	111.87 (12)	Fe—C9—H9	125.7
C6—Fe—C8	68.13 (12)	C9—C10—C6	107.4 (3)
C9—Fe—C8	39.76 (15)	C9—C10—Fe	70.02 (18)
C9^i^—Fe—C8	140.24 (15)	C6—C10—Fe	69.67 (18)
C7^i^—Fe—C8	139.95 (13)	C9—C10—H10	126.3
C7—Fe—C8	40.05 (13)	C6—C10—H10	126.3
C10^i^—Fe—C8^i^	67.98 (14)	Fe—C10—H10	125.6
C10—Fe—C8^i^	112.02 (14)	C12—C11—C16	119.4 (3)
C6^i^—Fe—C8^i^	68.13 (12)	C12—C11—P1	122.1 (2)
C6—Fe—C8^i^	111.87 (12)	C16—C11—P1	118.5 (2)
C9—Fe—C8^i^	140.24 (15)	C11—C12—C13	119.7 (3)
C9^i^—Fe—C8^i^	39.76 (15)	C11—C12—H12	120.1
C7^i^—Fe—C8^i^	40.05 (13)	C13—C12—H12	120.1
C7—Fe—C8^i^	139.95 (13)	C14—C13—C12	120.6 (3)
C8—Fe—C8^i^	180.0	C14—C13—H13	119.7
C1—S1—Au	98.40 (10)	C12—C13—H13	119.7
C6—P1—C17	105.75 (14)	C13—C14—C15	119.9 (3)
C6—P1—C11	105.61 (14)	C13—C14—H14	120.0
C17—P1—C11	103.37 (13)	C15—C14—H14	120.0
C6—P1—Au	108.05 (10)	C16—C15—C14	119.6 (3)
C17—P1—Au	115.04 (10)	C16—C15—H15	120.2
C11—P1—Au	118.03 (10)	C14—C15—H15	120.2
C1—N1—C2	124.6 (2)	C15—C16—C11	120.7 (3)
C1—N1—C5	123.5 (2)	C15—C16—H16	119.6
C2—N1—C5	111.5 (2)	C11—C16—H16	119.6
N1—C1—S2	121.7 (2)	C18—C17—C22	119.1 (3)
N1—C1—S1	116.5 (2)	C18—C17—P1	120.7 (2)
S2—C1—S1	121.79 (18)	C22—C17—P1	120.2 (2)
N1—C2—C3	103.7 (2)	C19—C18—C17	120.6 (3)
N1—C2—H2A	111.0	C19—C18—H18	119.7
C3—C2—H2A	111.0	C17—C18—H18	119.7
N1—C2—H2B	111.0	C20—C19—C18	119.3 (3)
C3—C2—H2B	111.0	C20—C19—H19	120.3
H2A—C2—H2B	109.0	C18—C19—H19	120.3
C4—C3—C2	104.7 (3)	C21—C20—C19	120.6 (3)
C4—C3—H3A	110.8	C21—C20—H20	119.7
C2—C3—H3A	110.8	C19—C20—H20	119.7
C4—C3—H3B	110.8	C20—C21—C22	120.2 (3)
C2—C3—H3B	110.8	C20—C21—H21	119.9
H3A—C3—H3B	108.9	C22—C21—H21	119.9
C3—C4—C5	104.1 (3)	C21—C22—C17	120.0 (3)
C3—C4—H4A	110.9	C21—C22—H22	120.0
C5—C4—H4A	110.9	C17—C22—H22	120.0
C3—C4—H4B	110.9		
			
C2—N1—C1—S2	−173.8 (2)	C8—C9—C10—Fe	60.3 (2)
C5—N1—C1—S2	−2.2 (4)	C7—C6—C10—C9	−0.2 (3)
C2—N1—C1—S1	6.1 (4)	P1—C6—C10—C9	178.2 (2)
C5—N1—C1—S1	177.7 (2)	Fe—C6—C10—C9	60.1 (2)
Au—S1—C1—N1	−164.2 (2)	C7—C6—C10—Fe	−60.3 (2)
Au—S1—C1—S2	15.7 (2)	P1—C6—C10—Fe	118.1 (3)
C1—N1—C2—C3	−179.5 (3)	C6—P1—C11—C12	−7.3 (3)
C5—N1—C2—C3	8.0 (3)	C17—P1—C11—C12	103.5 (3)
N1—C2—C3—C4	−27.0 (3)	Au—P1—C11—C12	−128.2 (2)
C2—C3—C4—C5	35.8 (3)	C6—P1—C11—C16	174.7 (2)
C1—N1—C5—C4	−158.8 (3)	C17—P1—C11—C16	−74.4 (3)
C2—N1—C5—C4	13.8 (3)	Au—P1—C11—C16	53.8 (3)
C3—C4—C5—N1	−30.3 (3)	C16—C11—C12—C13	−1.4 (5)
C17—P1—C6—C7	150.6 (2)	P1—C11—C12—C13	−179.3 (2)
C11—P1—C6—C7	−100.2 (3)	C11—C12—C13—C14	0.7 (5)
Au—P1—C6—C7	27.0 (3)	C12—C13—C14—C15	0.1 (5)
C17—P1—C6—C10	−27.5 (3)	C13—C14—C15—C16	−0.2 (5)
C11—P1—C6—C10	81.6 (3)	C14—C15—C16—C11	−0.5 (5)
Au—P1—C6—C10	−151.2 (3)	C12—C11—C16—C15	1.3 (5)
C17—P1—C6—Fe	63.9 (2)	P1—C11—C16—C15	179.3 (2)
C11—P1—C6—Fe	173.08 (17)	C6—P1—C17—C18	64.2 (3)
Au—P1—C6—Fe	−59.75 (19)	C11—P1—C17—C18	−46.6 (3)
C10—C6—C7—C8	−0.1 (4)	Au—P1—C17—C18	−176.7 (2)
P1—C6—C7—C8	−178.6 (2)	C6—P1—C17—C22	−113.7 (3)
Fe—C6—C7—C8	−59.7 (2)	C11—P1—C17—C22	135.5 (2)
C10—C6—C7—Fe	59.6 (2)	Au—P1—C17—C22	5.4 (3)
P1—C6—C7—Fe	−118.9 (2)	C22—C17—C18—C19	3.0 (4)
C6—C7—C8—C9	0.3 (4)	P1—C17—C18—C19	−175.0 (2)
Fe—C7—C8—C9	−58.4 (2)	C17—C18—C19—C20	−2.8 (5)
C6—C7—C8—Fe	58.7 (2)	C18—C19—C20—C21	0.7 (5)
C7—C8—C9—C10	−0.4 (4)	C19—C20—C21—C22	1.2 (5)
Fe—C8—C9—C10	−59.1 (2)	C20—C21—C22—C17	−1.0 (5)
C7—C8—C9—Fe	58.7 (2)	C18—C17—C22—C21	−1.0 (4)
C8—C9—C10—C6	0.4 (4)	P1—C17—C22—C21	176.9 (2)
Fe—C9—C10—C6	−59.9 (2)		

Symmetry code: (i) −*x*+1, −*y*, −*z*+2.

### Hydrogen-bond geometry (Å, º)

**Table:**

*D*—H···*A*	*D*—H	H···*A*	*D*···*A*	*D*—H···*A*
C13—H13···S2^ii^	0.95	2.86	3.680 (3)	144
C20—H20···S2^iii^	0.95	2.84	3.628 (3)	141

Symmetry codes: (ii) *x*, −*y*+1/2, *z*+1/2; (iii) −*x*+1, −*y*, −*z*+1.
